# Regulation of Immune Cell Infiltration into the CNS by Regional Neural Inputs Explained by the Gate Theory

**DOI:** 10.1155/2013/898165

**Published:** 2013-08-06

**Authors:** Yasunobu Arima, Daisuke Kamimura, Lavannya Sabharwal, Moe Yamada, Hidenori Bando, Hideki Ogura, Toru Atsumi, Masaaki Murakami

**Affiliations:** ^1^JST-CREST, Graduate School of Frontier Biosciences, Graduate School of Medicine, and WPI Immunology Frontier Research Center, Osaka University, Osaka 565-0871, Japan; ^2^Office for University-Industry Collaboration, Osaka University, Science Innovation Center Building A, 4th Floor, 2-1 Yamadaoka, Suita, Osaka 565-0871, Japan

## Abstract

The central nervous system (CNS) is an immune-privileged environment protected by the blood-brain barrier (BBB), which consists of specific endothelial cells that are brought together by tight junctions and tight liner sheets formed by pericytes and astrocytic end-feet. Despite the BBB, various immune and tumor cells can infiltrate the CNS parenchyma, as seen in several autoimmune diseases like multiple sclerosis (MS), cancer metastasis, and virus infections. Aside from a mechanical disruption of the BBB like trauma, how and where these cells enter and accumulate in the CNS from the blood is a matter of debate. Recently, using experimental autoimmune encephalomyelitis (EAE), an animal model of MS, we found a “gateway” at the fifth lumber cord where pathogenic autoreactive CD4+ T cells can cross the BBB. Interestingly, this gateway is regulated by regional neural stimulations that can be mechanistically explained by the gate theory. In this review, we also discuss this theory and its potential for treating human diseases.

## 1. Mechanism for BBB Breakdown in Autoimmunity of the CNS

**Figure 1:**
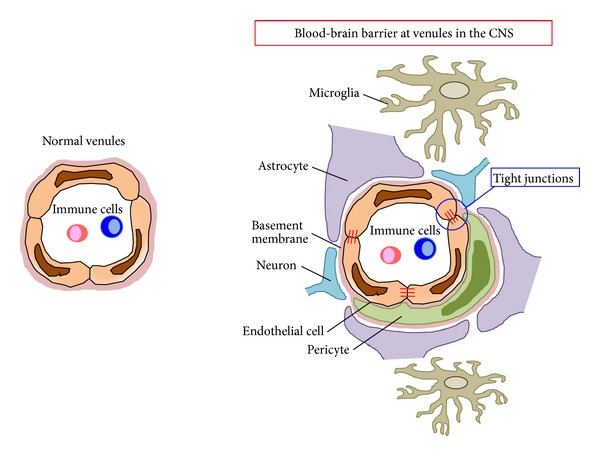
Venules at the blood-brain barrier in the CNS and other organs. Venules at the blood-brain barrier consist of specific endothelial cells that are brought together by tight junctions and tight liner sheets formed by pericytes and astrocytic end-feet. These are not present in normal venules (left).

The blood-brain barrier (BBB) in blood vessels is known to strictly limit the inflow of substances like proteins and cells from the bloodstream into the CNS (Figure 1), thereby maintaining a homeostatic environment for surrounding neurons and glia cells, a property different from that in peripheral organs. The BBB is formed and maintained by endothelial cells and corresponding tight junctions formed by claudins and occludins in collaboration with pericytes, microglial cells, macrophages, and astrocytes [1, 2]. BBB dysfunction is known to be associated with chronic neurodegenerative disorders, such as Parkinson's disease and Alzheimer's disease, and autoimmune diseases in the CNS [3, 4]. An increasing number of studies have shown that one cause of a dysfunctional BBB is inflammatory cytokines. For example, tumor-necrosis factor *α* (TNF*α*), interleukin-(IL-) 1*β*, and IL-17A have all been reported to loosen the BBB [5]. In particular, IL-17A is known to disrupt the BBB *in vitro *and *in vivo*. Huppert et al. reported that IL-17A-induced BBB dysfunction involves the formation of reactive oxygen species by NADPH oxidase and xanthine oxidase and that these species lead to the down regulation of tight junction molecules and the activation of the endothelial contractile machinery *in vitro* [6]. In addition, Kebir et al. reported that treatment with IL-17A increases the protein permeability of human brain endothelial cells and that this permeability is associated with a decrease in the expression of occludin and ZO-1 [7]. A role of IL-17A in BBB disruption has also been found using experimental autoimmune encephalomyelitis (EAE) mice, an animal model of multiple sclerosis (MS), *in vivo*. In this model, the major source of IL-17A is type-17 helper T (Th17) cells, particularly autoreactive ones. EAE is significantly suppressed in IL-17A-deficient mice. Instead, these mice exhibit delayed onset, reduced maximum severity scores, ameliorated histological changes, and early recovery [8]. Additionally, an adoptive transfer model in which helper T cells obtained from myelin oligodendrocyte glycoprotein (MOG) immunized mice were infused into naïve recipients indicated that IL-17A derived from CD4+ T cells is critical for the induction of EAE [8]. In addition, MOG-reactive Th17 cells obtained from MOG-immunized IL-17A-deficient mice were unable to infiltrate the lumber level of spinal cord in the same model (see the following for details) [9]. Furthermore, it was shown that the adoptive transfer of Th17 cells from ovalbumin-specific T cell receptor transgenic mice, which are unable to recognize CNS antigens, does not pass the BBB and migrate into CNS, whereas cotransfer of these Th17 cells with MOG-reactive Th17 cells leads to the accumulation of both types of Th17 cells in the CNS (our unpublished data and [10]), which strongly suggests that antigen recognition of Th17 cells is required for severe disruption of the BBB. Although antigen presentation inside the CNS has suggested through observation that the infusion of ovalbumin peptide-loaded antigen-presenting cells into cerebrospinal fluids induces an accumulation of ovalbumin-specific Th17 cells in the CNS [10], the type of antigen-presenting cells and location where antigen presentation takes place under physiological conditions remain elusive. Nevertheless, these results suggest that IL-17A expressing Th17 cells, which recognize CNS antigens, have a major impact on breaching the BBB, in part by decreasing tight junction molecules. 

## 2. Neuroimmune Interactions Responsible for Inflammation in the CNS

In the previous section, we discussed the relationship between inflammatory cytokines such as IL-17A and disruption of the BBB. This section focuses on the gateway for which pathogenic CD4+ T cells enter the CNS. In patients with MS, common early symptoms include vision problems and tingling, followed by many neurological signs as the disease progresses. It is known that inflammation sites in MS are found in specific regions of white matter, including the brainstem, the optic nerve, the cerebellum, the long motor, and sensory tracts of the spinal cord [11]. This fact suggests that some CNS regions might be more vulnerable to autoimmune attacks. One hypothesis proposes that chemokine recruitment of pathogenic autoreactive T cells is more abundant in these regions. Among the many chemokines, *CCL20* is of particular interest, as it attracts Th17 cells that express CCR6, a receptor for CCL20. Reboldi et al. reported that mice lacking CCR6 are highly resistant to EAE and that the choroid plexus, a specialized epithelial structure in the brain known to produce cerebrospinal fluids, expresses CCL20 constitutively, an effect that acts as an attractant for the first wave of CCR6+ Th17 cells [12]. In that same study, however, EAE was induced using the complete Freund's adjuvant, which is widely used to generate active immunization in animals but is also an inducer of systemic inflammation and has many side effects including fever, motor neuron dysfunction such as paralysis, and apoptosis. These side effects could affect the pathophysiological status of the brain and spinal cords resulting in different conclusions from the steady state. 

**Figure 2:**
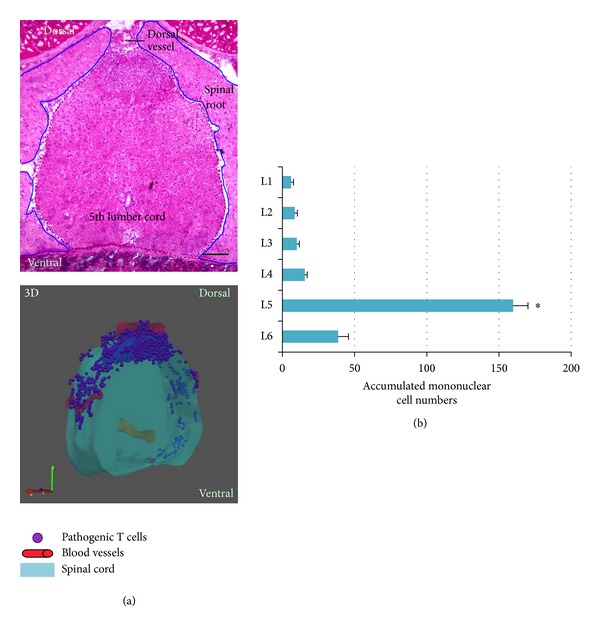
The fifth lumber cord is a gateway into the CNS. A cross section of the fifth lumber (L5) cord (a) and actual cell numbers of mononuclear cells accumulated in each lumber cord segment (b) at a preclinical phase of EAE (5 days after pathogenic Th17 transfer). A 3D picture based on ten serial sections of L5 is also shown.

We recently found a “gate” past the BBB in the spinal cord that autoreactive Th17 cells in the bloodstream can exploit to enter the CNS. To make this discovery, we first utilized an adoptive transfer model to induce EAE in which Th17 cells obtained from MOG-immunized mice were infused into naïve recipient mice to maintain CNS quiescence. In this adoptive transfer model, we found that MOG-reactive Th17 cells preferentially accumulated in the fifth lumber (L5) cord rather than the brain or other levels of the spinal cords at the earliest phase of EAE (day 5 after T cell transfer) [9] (Figure 2). This finding fits well with a typical clinical EAE sign in which the tail is first affected. We also found that blood vessel tracks in L5 are altered due to the formation of edema in the L5 cord by using a supersensitive MRI (data not shown). Consistent with these results, *Ccl20* mRNA levels were highest in the dorsal venules of L5 compared with those from other spinal cords, and the transfer of CCR6-deficient Th17 cells did not accumulate in the L5 region. Interestingly, even in naïve animals without Th17 transfer, mRNA levels of *Ccl20* and many other chemokines were specifically upregulated in the dorsal venules of L5. Therefore, dorsal venules in the L5 spinal cord have special properties in diseased as well as healthy conditions. 

**Figure 3:**
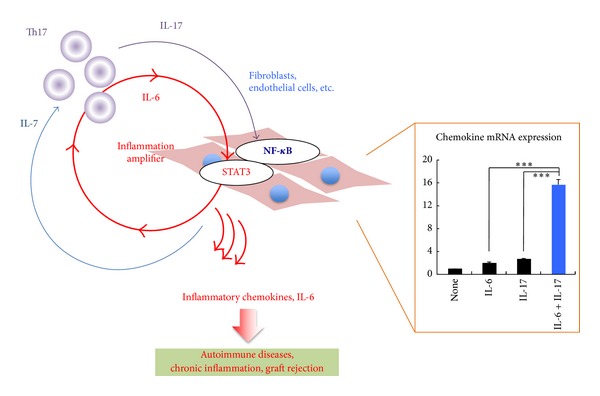
The inflammation amplifier is defined as a mechanism that triggers the hyperinduction of cytokines and chemokines in nonimmune cells and leads to many diseases. This induction is induced by the simultaneous activation of NF-*κ*B and STAT3. IL-7 from nonimmune cells also contributes to enhancing the inflammation amplifier by generating Th17 cells and/or sustaining their survival. The inflammation amplifier is essential for the pathogenesis of F759 arthritis, EAE, and chronic graft rejection. The bar graph on the right indicates typical expression statuses of chemokines and IL-6 after inflammation amplifier activation (see the blue bar).

We previously found a mechanism for the hyperinduction of inflammatory chemokines and cytokines in nonimmune cells such as fibroblasts, endothelial cells, and epithelial cells using a rheumatoid arthritis model. The mechanism is driven by a simultaneous activation of two transcription factors, NF-*κ*B and STAT3. Thus, it was named the “inflammation amplifier”, because hyperactivation of NF-*κ*B by activated STAT3 induces large amounts of NF-*κ*B-targeted chemokines and chemotactic factors to promote the recruitment of immune cells (Figure 3). *Ccl20* is one such target chemokine and is found in vascular endothelial cells. Given that chemokine expressions are elevated in L5 dorsal venules, we hypothesized a role for the inflammation amplifier. The activation status of  NF-*κ*B and STAT3 is indeed higher in L5 dorsal venules than other lumber cords even in naïve healthy mice. The elevated *Ccl20* mRNA levels at L5 vessels were decreased in mice devoid of the inflammation amplifier such as IL-6-deficient mice and endothelial cell-specific IL-6 receptor (gp130) deficient mice. Even under healthy conditions, it is known that some immune cells are present in the CNS, suggesting that there may be a gate to enter the restricted tissue regardless. In this respect, it is tempting to speculate that low-grade activation of the inflammation amplifier at L5 dorsal venules creates the gate by inducing certain levels of chemokines, although further studies are required for direct evidence that links activation of the inflammation amplifier with immunity homeostasis in the CNS. 

**Figure 4:**
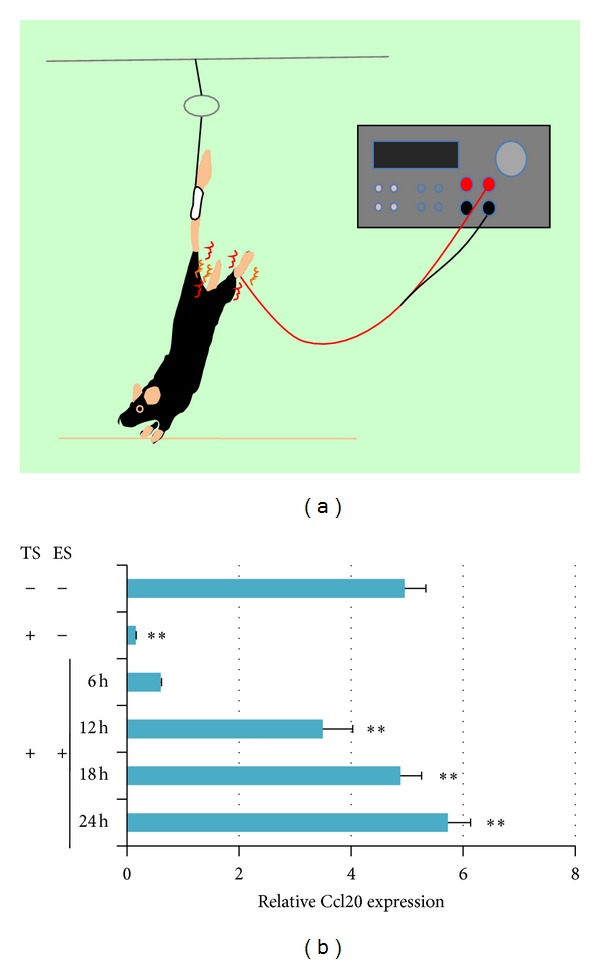
Neural stimulation-mediated activation of the inflammation amplifier creates a gateway into the CNS via chemokine production. Schematic illustration of the tail suspension model (a). A string is fastened to the roof of the cage at a height that allows the forelimbs to support body weight but prevents the hindlimbs from touching any part of the cage. Release from gravitational stimuli caused by tail suspension (TS) results in a decrease of Ccl20 levels at the L5 dorsal venules. Electric stimulation (ES) during TS restores the levels in a time-dependent manner (b).

Upon discovering the L5 cord as the entry site of autoreactive Th17 cells at the initial phase of EAE [9], we searched for reasons that would make L5 ideal for this gate. The answer came from physiological responses to gravitation stimuli. Soleus muscles are constantly stimulated by gravitational forces, and the dorsal root ganglia of their sensory neurons are located beside the L5 cord [13]. We hypothesized that frequent stimulation of the soleus muscles by gravity could induce activation of the inflammation amplifier via sensory nerves. In experiments that had healthy normal mice suspended from their tails so that only the forelimbs could touch the ground and the hind legs were released from gravitational forces, MOG-reactive Th17 cells no longer accumulated at L5 (Figure 4). Instead, they accumulated at cervical cords, indicating that burdening the arm muscles with body weight opened a new gateway for immune cells [9]. Consistent with this observation, tail suspension significantly inhibited *Ccl20* mRNA expression in L5 dorsal blood vessels and decreased the expression of the neural activation marker, c-Fos, in L5 dorsal root ganglia. In addition, when the soleus muscles of tail-suspended mice were artificially stimulated by weak electric pulses, *Ccl20* expression, MOG-specific Th17 accumulation, and c-Fos levels were restored at L5 (Figure 4(b) and not shown). These data strongly suggest that neural activation by an antigravitational response plays a role in the activation of the inflammation amplifier, leading to the expression of many chemokines including Th17-attracting *Ccl20* in L5 dorsal blood venules [9]. 

**Figure 5:**
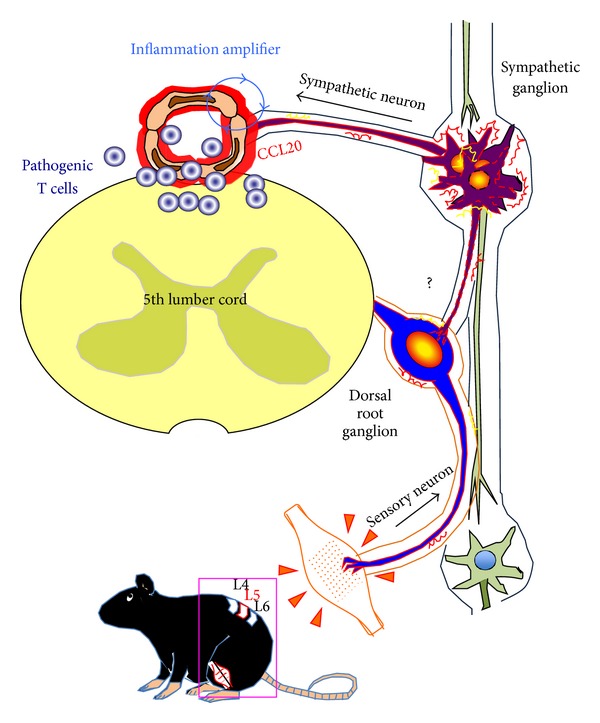
Schematic representation of neural stimulation-mediated activation of the inflammatory amplifier. Neural signals from the soleus muscles caused by gravitational stimulation reache the L5 dorsal root ganglion. Subsequent activation of sympathetic nerves alters the status of endothelial cells in L5 dorsal venules to enhance the inflammation amplifier, which leads to the production of chemokines including CCL20. Norepinephrine acts as a mediator between the neural signal and activation of the inflammation amplifier. The neural network determining communication between soleus muscle-derived sensory neurons and sympathetic neurons that reach L5 is not currently defined (depicted with a question mark).

What mechanisms do afferent sensory neurons from the soleus muscle use to regulate the status of blood venules at L5? Although a precise neural network remains unclear, we have shown sympathetic nerves to be involved. Blood flow speed at L5 dorsal venules became slower when mice are tail suspended, while electronic stimulation of the soleus muscles increases the flow, suggesting that automatic nerves including sympathetic ones are involved in the response. On the other hand, blood flow speeds in blood vessels other than the L5 region, such as femoral vessels, brain surface vessels, and the portal vein, are not affected by tail suspension. Furthermore, treatment with atenolol, a *β*1 adrenergic receptor antagonist, or prazosin, an *α*1 adrenergic receptor antagonist, significantly suppresses *Ccl20* mRNA expression, NF-*κ*B activation, and MOG-reactive Th17 accumulation at L5 vessels and also suppresses clinical signs of EAE [9]. Consistent with these *in vivo* results, the addition of norepinephrine to a culture of endothelial cell lines enhances the inflammation amplifier based on IL-6 and Ccl20 expressions. Thus, anti-gravity responses of the soleus muscles result in sympathetic nerve stimulation, which creates a gateway of immune cells to pass though the CNS via L5 dorsal venules [9]. Based on these findings, we propose that MOG-reactive, disease-causing Th17 cells make use of the L5 gateway to infiltrate the CNS and induce local inflammation by producing cytokines like IL-17A, which further induces chemokines through the inflammation amplifier and results in chronic inflammation of the CNS (Figure 5). At a later phase of EAE, transferred Th17 cells are also found in the brain including regions like the choroid plexus and cerebellum. Whether these Th17 cells move within the cerebrospinal fluid to finally reach the brain and/or enter the brain directly from the circulation is unclear. However, in the brain of EAE mice with advanced clinical scores, transferred Th17 cells are not uniformly localized. Rather, they tend to accumulate in specific areas of the brain (our unpublished data), which argues for the involvement of neural activation and subsequent breach of the BBB in these areas. Using this logic, we speculate that the relatively high incidence of vision dysfunction during the initial phases of MS in humans might be due to the persistent visual stimulation in our everyday lives that activates the optical nerves and unlocks the gate nearby. 

Other neuroimmune interactions have been reported by other groups. The Kevin Tracey group, which is a pioneer in this field, has demonstrated that vagus nerve stimulation suppresses the release of proinflammatory cytokines through the nicotinic acetylcholine receptor *α*7 subunit and identified a subset of T cells producing acetylcholine that can relay neural signals [14–16]. Acetylcholine is also produced by other immune cells including B cells, which have an impact on innate immunity [17]. Cao et al. reported that mice reared in a larger cage with toys and more mice, that is, an enriched environment, are resistant to tumor burden in a manner dependent on sympathetic nerve activation via the BDNF/leptin axis [18]. Nguyen et al. reported a relationship between catecholamines, alternative macrophages, and thermogenesis, finding that exposure to cold temperatures rapidly promotes alternative activation of adipose tissue macrophages, leading to a secretion of catecholamines that induces thermogenic gene expressions in brown adipose tissue and lipolysis in white adipose tissues [19]. Additionally, Hassan et al. showed that behavioral stress promotes prostate cancer development by inhibiting the apoptosis of tumor cells via the *β*2-adrenergic receptor [20]. Therefore, a strategy to modulate neuroimmune interactions may prove a promising approach for therapeutic interventions against many inflammatory diseases. 

## 3. The Gate Theory

**Figure 6:**
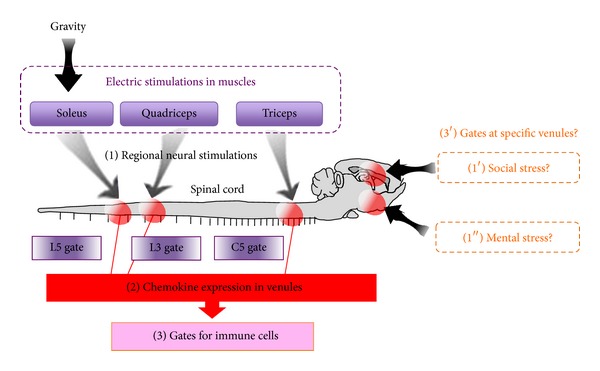
Each neural stimulation establishes a regional gate of immune cells in the CNS. Gravity or electric stimulations to soleus muscles can open the L5 gateway via regional neural stimulations-mediated chemokine expression, electronic stimulation of the quadriceps followed by neural stimulation establishes the L3 gateway, and gateways in the fifth cervical to fifth thoracic cord vessels were created by electric stimulations to triceps brachii. Therefore, it is reasonable that neural stimulations induced by stresses like social and mental ones might establish gates in some specific sites of the brain.

**Figure 7:**
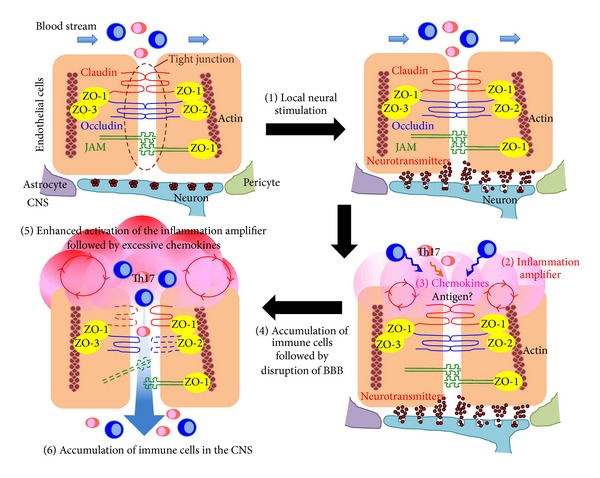
Schematic representation of the gate theory in the CNS. The gate theory describes how regional neural stimulations direct immune cell infiltration into target organs by crossing gates located at various blood vessels. Immune cells in the blood stream cannot enter the target organs due to homeostasis of the venules (upper left). Regional neural stimulation secretes neurotransmitters including norepinephrine into the endothelial cells of the venules (upper right). This stimulation activates the inflammation amplifier in the endothelial cells followed by increasing local chemokine expression (bottom right). The accumulation of immune cells disrupts the BBB, which allows immune cells to enter the CNS (bottom left).

The dorsal venules of the L5 spinal cord have been found to act as a gateway for MOG-reactive Th17 cells to accumulate into the CNS in an adoptive transfer model of murine EAE during steady state. Gravity or electric stimulations to soleus muscles can open the L5 gateway, as described above. We extended these findings by applying electric pulses to other muscle regions. Electronic stimulation of the quadriceps or thigh muscles, which are known to be regulated by L3 dorsal-root ganglion neurons, increased the expression of *Ccl20* mRNA in L3 cord vessels in mice. Similarly, chemokine levels in the fifth cervical to fifth thoracic cord vessels were upregulated by electric stimulations to epitrochlearis/triceps brachii (upper arm muscles), which are controlled by neurons located at the corresponding spinal regions (Figure 6) [9]. Based on these findings, we proposed “the gate theory”, which describes how regional neural stimulations direct immune cell infiltration into target organs by crossing gates located at various blood venules (Figure 7). Investigations on whether the gate theory can be applied to tissues other than the CNS are ongoing. The ability to manipulate these gates at targeted regions in the body is expected to have significant clinical benefits, as closing them should ameliorate autoimmune inflammation in the target organ without any systemic immune suppression, while opening these gates near surrounding tumors may enhance cancer immunotherapy effects. With such medical promise, much effort is needed to identify the precise molecular mechanisms for gating.
